# Understanding Olive Oil Stability Using Filtration and High Hydrostatic Pressure

**DOI:** 10.3390/molecules25020420

**Published:** 2020-01-20

**Authors:** Lorenzo Guerrini, Bruno Zanoni, Carlotta Breschi, Giulia Angeloni, Piernicola Masella, Luca Calamai, Alessandro Parenti

**Affiliations:** Dipartimento di Scienze e Tecnologie Agrarie, Alimentari, Ambientali e Forestali (DAGRI), Università degli Studi di Firenze, Piazzale delle Cascine 15, 50144 Florence, Italy; bruno.zanoni@unifi.it (B.Z.); carlotta.breschi@unifi.it (C.B.); giulia.angeloni@unifi.it (G.A.); piernicola.masella@unifi.it (P.M.); luca.calamai@unifi.it (L.C.); alessandro.parenti@unifi.it (A.P.)

**Keywords:** biophenols, microbial contamination, sensory defects, turbidity, water content

## Abstract

Veiled extra virgin olive oil (VEVOO) is very attractive on the global market. A study was performed to highlight the role of different amounts of water and microorganisms on the evolution of VEVOO quality during storage, using the selective effects of the application of individual or combined filtration and high hydrostatic pressure (HHP) treatments. Four oil processing trials were carried out in four replicates, resulting in a full factorial design with two independent fixed factors: filtration and HPP treatments. The turbidity of all the olive oil samples was characterized. Furthermore, all the olive oil samples were analysed for legal parameters, volatile organic compounds and phenolic compounds during the storage tests. The microbial contamination in the presence of a high level of water activity (>0.6 Aw) was related to the formation of volatile aroma compounds, which were responsible for the “fusty” sensory defect. Furthermore, high water activity values were related to an increase in the hydrolytic degradation rate of the phenolic compounds. The oil turbidity has to be planned and controlled, starting from adjustment of the water content and application of good manufacturing practices.

## 1. Introduction

Extra virgin olive oil (EVOO) is considered a food with a long shelf life. However, during storage EVOO undergoes several compositional changes that reduce its quality. These changes can affect both the chemical and sensory criteria that must be met for the European legal classification of EVOO as well as its nutritional value.

With respect to the European legal requirements [1], the most frequently considered parameters are the amount of free fatty acids (i.e., the acidity value), peroxide values and UV index (i.e., K232, K270 and ΔK) values, in order to evaluate the level of enzymatic hydrolysis and radical oxidation of the triacylglycerols, respectively. EVOO must also have both a minimum positive “fruity” attribute and no negative sensory attributes (i.e., defects). Panel testing is the official method to measure the above attributes, even though some relationships have been reported in the literature between sensory perception and volatile organic compound content [2,3].

EVOO is rich in phenolic compounds, which are natural antioxidants with several positive effects on human health, playing a role in preventing several diseases [4]. The beneficial effect of phenolic compounds from olives has been confirmed by the following scientific opinion from the European Food Safety Authority (EFSA) in relation to permitted health claims [5,6,7]: “Olive oil polyphenols contribute to the protection of blood lipids from oxidative stress”.

Veiled extra virgin olive oil (VEVOO) is described as a dispersion-suspension system, with the degree of turbidity resulting from the presence of micro-droplets of vegetation water and small solid fragments of olive skin and pulp covered by a film of water [8,9]. VEVOO is always very attractive on the global market, since for many consumers VEVOO is deemed to be of a higher quality than filtered extra virgin olive oil (FEVOO). However, this subject is still controversial.

Some literature data have shown a lower level of radical oxidation of the triacylglycerols in VEVOO than in FEVOO; the resulting increase in shelf life was explained by the higher content of antioxidant phenolic compounds such as secoiridoids (i.e., oleuropein, ligstroside and their derivatives) in VEVOO, since they are not removed by filtration [10,11,12,13,14]. Instead, other literature data have shown VEVOO to have a higher risk of degradation during shelf life than FEVOO; the water content, combined with the spoilage microorganisms (i.e., mainly yeasts) which are contained in the micro-droplets of water and the solid particles [15], was related to both an increase in secoiridoid degradation and the development of sensory defects in VEVOO [16,17,18,19]. Finally, other literature data have shown no significant qualitative differences during shelf life between VEVOO and FEVOO [8,20].

Therefore, a study on the effects of chemical and microbial transformation phenomena on VEVOO quality may be useful; an explanation of the potential different roles between the water and solid particle contents and microbial contamination may be particularly interesting too. In this work, the selective effects of the application of individual or combined filtration and high hydrostatic pressure (HHP) treatments were tested. Indeed, filtration is able to remove water and solid particles [21], while HHP is able to inactivate microorganisms [22]. In this way, a study was performed of the role of different amounts of water and microorganisms on the evolution of EVOO quality during storage. Four oil processing trials were carried out, resulting in a full factorial design with four specimens: (i) CON (i.e., no filtration and no HHP), (ii) FIL (i.e., filtration and no HHP), (iii) HHP (i.e., no filtration and HHP), (iv) F-HHP (i.e., filtration and HHP). All of the olive oil samples were analysed to measure some turbidity characterization parameters (i.e., degree of turbidity, water content, water activity, solid particle content, microbial cell count) and the EVOO legal requirements, the volatile organic compounds and the phenolic compounds during storage tests. The storage conditions were chosen to potentially cause the transformation phenomena on olive oil samples quality.

## 2. Results

### 2.1. Effect of Treatments on Turbidity Characterization

The filtration and HHP treatments had a significant effect on the turbidity characterization parameters of the just processed olive oil samples (Table 1).

All the veiled oil samples (i.e., CON and HHP samples) showed a high degree of turbidity (approx. 1500 NTU), since they were collected at the end of the “decanter” without having undergone any preliminary centrifugation or decantation treatments [18,21,23]. Consistently, the CON and HHP samples had high water (0.25% *w*/*w*) and solid particle content values (0.22% *w*/*w*) and high levels of water activity (0.76 A_W_). The CON samples were also contaminated by microorganisms with microbial counts in the range of 4–5 log UFC/g, which may be related to the sanitary conditions of the olive fruits and the hygiene conditions of the olive oil mill [13,15,24]. The HHP treatment was able to inactivate the microorganisms; the HHP samples contained no microorganisms, even though the values of the other turbidity characterization parameters remained the same as the CON samples. All of the filtered oil samples (i.e., FIL and F-HHP samples) showed a low degree of turbidity (15 NTU), a low water content (0.05% *w*/*w*), no solid particle content and low water activity values (0.42 A_W_). The separation of water and solid particles by filtration also caused the complete removal of microorganisms.

### 2.2. Quality Evolution During Storage

#### 2.2.1. European Legal Requirements

All of the olive oil samples were compliant with the EU legal chemical limits [1] during the storage tests (Supplementary Material Table S1); no significant variations occurred between either the CON, HHP, FIL and F-HHP samples or the different storage times (i.e after 15 days, 1 month and 6 months of storage). The oil samples had low acidity values (approx. 0.21% oleic acid), low peroxide values (approx. 4.9 meq O_2_ kg^−1^) and low UV index values (approx. 1.67 and 0.13 for K_232_ and K_270_, respectively). A color shift from green to yellow was visually noticed in all samples regardless the treatment, probably due to the light effect on samples.

Instead, the filtration and HHP treatments had a significant effect on the legal sensory attributes of the olive oil (Table 2). The positive “fruity” attribute showed a significant change as a function of filtration and storage time. The filtered oil (FIL and F-HHP samples) was perceived as fruitier (*p* < 0.001) than the veiled oil (CON and HHP samples). Furthermore, in all of the olive oil samples the fruitiness attribute significantly decreased during storage (*p* < 0.001). The positive bitterness attribute changed significantly as a function of filtration (*p* < 0.01), storae time (*p* < 0.001) and their interaction (*p* < 0.05). The filtered oil was bitterer than the veiled oil after 15 days of storage, while no significant differences occurred after 1 and 6 months of storage; the bitterness significantly decreased in intensity during storage. The behaviour of the positive pungency attribute during storage was also consistent with the bitterness attribute.

“Fusty” and “rancid” sensory defects occurred in some olive oil samples, causing the oil to be downgraded from EVOO to virgin or lampante olive oil. The negative “fusty” attribute was related to filtration (*p* < 0.001), HHP treatment (*p* < 0.05) and their interaction (*p* < 0.01). The CON oil samples were the only ones with the “fusty” defect. The negative “rancid” attribute was significantly related to the treatments, storage time and all of their interactions. The “rancid” defect was not present in the filtered oil samples during storage, but it was perceived in the veiled oil samples after 1 month of storage. The intensity of the rancidity attribute was high and increased with storage time in the CON oil samples.

#### 2.2.2. Volatile Organic Compounds

The volatile organic compound content of the oil samples was studied as the three following groups of compounds, in relation to their assumed role in oil sensory quality: (i) compounds with five and six carbon atoms, which are usually associated with the lipoxygenase (LOX) pathway and, consequently, with the “fruity” and “green” positive sensory attributes [25]; (ii) microbial metabolite compounds, which are usually associated with negative sensory attributes such as “fusty”, “muddy”, “vinegary” and “mouldy” defects [2,15,17]; (iii) compounds with seven, eight and nine carbon atoms, which are usually associated with the “rancid” negative sensory attribute [2,3]. The list of the above measured compounds is presented as Supplementary Material in Table S2. The experimental data were processed as the sum of the three above groups of volatile organic compounds, except for the (*E*)-2-hexenal compound, which was certainly associated in the literature with the “fruity” and “green” positive sensory attributes (Table 3).

Data from all of the oil samples showed that C_6_ compounds from linolenic acid were the most abundant, in agreement with the literature [25,26], demonstrating that the LOX pathway had a preferential action on linolenic acid. (*E*)-2-Hexenal was consistently the most abundant compound (i.e., from 85% to 92% of the sum of C_6_ compounds).

The sum of the LOX compound content with five carbon atoms was not significantly related to filtration, HHP treatment or storage time, while sum of the LOX compound content with six carbon atoms significantly decreased during storage (*p* < 0.05). The (*E*)-2-hexenal content also showed a significant change as a function of filtration and storage time. The filtered oil (FIL and F-HHP samples) had a significantly (*p* < 0.01) higher content of (*E*)-2-hexenal than the veiled oil (CON and HHP samples). Furthermore, in all of the olive oil samples the (*E*)-2-hexenal content significantly decreased during storage (*p* < 0.001).

The sum of the microbial metabolite compound content was significantly influenced by the interaction between the filtration and HHP treatments (*p* < 0.05). Low contents of the above compounds were measured both in the filtered oil (FIL and F-HHP samples) and in the veiled oil treated with HHP (HHP samples), while the veiled oil (CON samples) had the highest content of microbial metabolite compounds.

The sum of the C_7_, C_8_, C_9_ and C_10_ compounds (i.e., the “rancid” compounds) showed significant differences as a result of filtration (*p* < 0.01), HHP treatment (*p* < 0.01) and interaction between filtration and HHP treatment (*p* < 0.01). After 15 days of storage, a higher content of the “rancid” compounds was measured in the veiled oil (CON samples) than in both the filtered oil (FIL and F-HHP samples) and the veiled oil treated with HHP (HHP samples). The above difference between the oil samples was lost after 1 and 6 months of storage.

#### 2.2.3. Phenolic Compounds

The phenolic compound content of the oil samples was studied as total content, content of groups of secoiridoid compounds and content of single representative secoiridoid compounds in EVOO [27]. The R-Index was also considered as described in the Materials and Methods section. Briefly, the R-index is the ratio between tyrosol+hydroxytyrosol and the total secoridoids content. It can be considered a useful indicator of the hydrolysis of secoiridoids.

The experimental conditions and their interaction had no significant effect on the total phenolic compound content, but the profile of the phenolic compounds changed significantly (Table 4).

The sum of the content of oleuropein and its derivatives, and, accordingly, the 3,4-DHPEA-EDA content showed a significant difference as a function of filtration treatment (*p* < 0.001). After 15 days of storage the veiled oil samples had lower contents of the above compounds than the filtered ones; this difference remained constant after 1 and 6 months of storage.

The hydroxytyrosol and tyrosol contents changed significantly as a function of filtration (*p* < 0.001), storage time (*p* < 0.001) and their interaction (*p* < 0.001). In particular, the above compound contents increased in the veiled oil samples during storage, while they remained approximately constant in the filtered oil samples. In the same way, the R-Index was significantly related to filtration, storage time and their interaction. The filtration treatment caused a decrease in the hydrolytic status of the secoiridoids, while the storage time caused an increase in the R-Index. The interaction between storage time and filtration highlighted that the veiled oil samples were the samples most susceptible to secoiridoid hydrolytic degradation during storage.

## 3. Discussion

According to the literature data [21,28,29], the applied filtration and HHP treatments were able to create olive oil samples with different microbial contamination, water content and water activity levels (Table 1). Therefore, this work achieved its aim of creating olive oil samples with different susceptibilities to microbial, enzymatic and non-enzymatic transformation phenomena [30]. The CON oil samples were highly susceptible to all the above phenomena, since they had a high level of microbial contamination, water content and water activity. Indeed, water activity values > 0.6 A_w_ potentially make foods more prone to transformation phenomena [31].

The HHP oil samples were highly susceptible to enzymatic and non-enzymatic phenomena only, since they had no microbial contamination, but a high water content and level of water activity. The FIL and F-HHP oil samples were not very susceptible to any of the above phenomena, since they had no microbial contamination, a low water content and low water activity (< 0.6 A_w_).

The evolution of the measured EU legal chemical limits during storage showed that neither enzymatic hydrolysis by lipases nor radical oxidation of the triacylglycerols occurred on any of the olive oil samples. The potential lipases from microorganisms [15] were not active, since the acidity value did not change in the CON samples. The potential endogenous lipases were not active either, since the acidity value did not change in the HHP samples. The relatively short storage time may explain the above behaviour; Fregapane et al. [16] observed a hydrolysis of triacylglycerols in unfiltered oil samples, but they were working under accelerated storage conditions at 40 °C in the dark.

No effect of water content or water activity was evidenced on the rate of radical oxidation of the triacylglycerols. The relatively short storage time may explain the above phenomenon, but contradictory literature data have suggested that water has a protective effect against oxidation [14,32] or that the rate of lipid oxidation is lowest at a water activity of 0.2–0.4 Aw [33]. However, Brkic Bubola et al. [34] also showed no significant effect between the oxidation levels of filtered and unfiltered olive oil.

Instead, the veiled oil samples were affected by significant changes of sensory attributes, and volatile organic and phenolic compound contents, which can be explained by the experimental data as an effect of either a microbial contamination or a high level of water activity.

**Figure 1:**
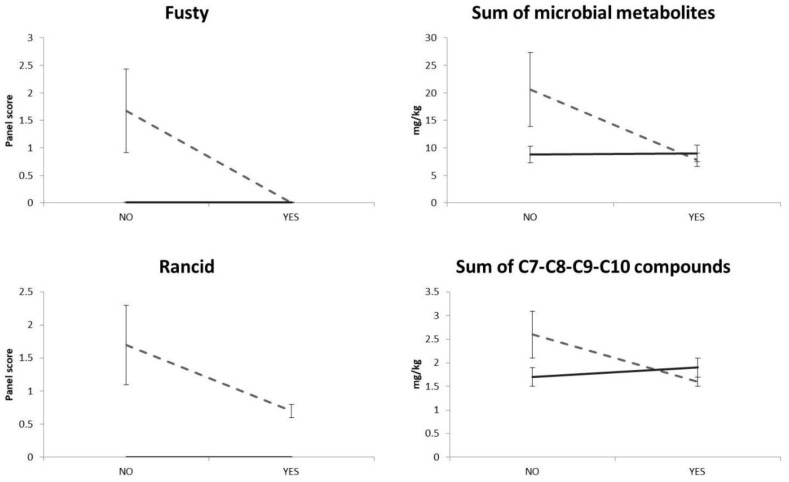
Interaction plots of “fusty” and “rancid” results given by the panel compared with chromatograph results (i.e., microorganism-related compounds for fustiness and C7-C8-C9-C10 compounds for rancidity). The *x*-axis reports HHP treatment (No or Yes). The continuous grey line shows filtered samples (i.e., filtration YES), and the black line cloudy samples (i.e., Filtration NO). Error bars represent the standard error.

Only the CON oil samples (i.e., with a high level of microbial contamination, water content and water activity) had a “fusty” defect during storage (Table 2) and an increasing content of microbial metabolite compounds during storage (Table 3). These behaviours can be considered congruent, related to each other and in line with some literature data [15,35]; Figure 1 clearly shows that the removal of microorganisms by filtration and HHP treatments prevented the formation of volatile organic compounds, which were responsible for the “fusty” defect.

Only the CON oil samples had a rancidity defect, which increased during storage (Table 2). Therefore, microbial activity may be related to the formation of the above sensory defect; this phenomenon, even though not well studied, has already been reported by both Guerrini et al. [24] and Ciafardini and Zullo [15], who linked the rapid appearance of the “rancid” defect with olive oil samples contaminated by yeasts. In this way, the appearance of the rancidity defect without a significant radical oxidation of the triacylglycerols in all the olive oil samples (as reported above in the text) may be explained. The experimental data relating to the C7, C8, C9 and C10 compound contents (i.e., the “rancid” compounds) appeared to be congruent with the above phenomenon, their highest content being in the CON oil samples (Table 3); the relationship between the “rancid” compounds and the same rancidity sensory defect is shown clearly in Figure 1.

The FIL and F-HHP oil samples (that is, with no microbial contamination, low water content and low water activity) were perceived by the panel test as fruitier than the veiled oil samples during storage (Table 2). This behaviour can be related to the LOX pathway. Indeed, it is known that during extraction processing both the olive oil fruits and the olive oil are subjected to the LOX pathway [36], which is the multi-step enzyme oxidation of linoleic and linolenic fatty acids into aldehydes, alcohols and esters with five and six atoms of carbon, responsible for pleasant sensory descriptors, such as “fruity” and “green” [25]. A common marker of the LOX pathway extent is E-2-hexenal, which was in fact the most abundant compound in all of the olive oil samples in this study (Table 3). A transformation of the LOX compounds can occur after oil extraction and during storage with a consistent decrease in the fruitiness attribute [16,20]. This transformation was evidenced by our experimental data: a decrease in (*E*)-2-hexenal content occurred and, consequently, the fruitiness attribute decreased (Table 2 and Table 3). Since the veiled oil samples displayed the greatest decrease in E-2-hexenal, it may be supposed that a high level of water activity has an effect on the increase in the LOX compound transformation rate; similar results were also reported by Fortini et al. [18]. Moreover, the above transformation may be caused by enzymatic or non-enzymatic reactions, without the involvement of microbial activity.

The FIL and F-HHP oil samples were perceived by the panel test as bitterer and more pungent than the veiled oil samples during storage (Table 2). This behaviour can be related to the phenolic profile of olive oil, which is not the same as the phenolic profile of olive oil fruits, since numerous transformation phenomena can occur during EVOO extraction processing and storage [37,38,39]. Since secoiridoids are the phenolic compounds with the highest transfer rate from fruits to oil, the predominant phenolic compounds in olive oil are oleuropein, ligstroside and their derivatives. Oleuropein and ligstroside are thought to be subjected to transformation, resulting in hydrolytic and oxidative changes of both an enzymatic and non-enzymatic nature. The hydrolytic transformation pathway causes the rapid formation of aglycones (3,4-DHPEA-EA - oleuropein aglycone; *p*-HPEA-EA - ligstroside aglycone), as a result of the hydrolysis of a sugar molecule, which can be caused by β-glucosidase activity. The obtained aglycones can undergo isomerization to open dialdehydic forms. Dialdehydic forms in turn decarboxylate into the respective aglycones (3,4-DHPEA-EDA - dialdehydic form of decarboxymethyl oleuropein aglycone; p-HPEA-EDA - dialdehydic form of decarboxymethyl ligstroside aglycone). 3,4 DHPEA-EDA is often EVOO’s most abundant phenolic compound. Finally, the compounds hydroxytyrosol (3,4-DHPEA) and tyrosol (*p*-HPEA) are formed slowly by hydrolysis of the ester linkage. The content of oleuropein, ligstroside and their derivatives was usually proportionally related to the intensity of bitterness and pungency and the positive effects of EVOO on human health [30]. The oxidative degradation of secoiridoids may follow both an enzymatic and a non-enzymatic degradation pathway. In the former pathway, polyphenol oxidases (PPO) and peroxidases (POD) catalyse the oxidation of phenolic compounds to corresponding quinones [40]. In the latter pathway, which is connected to termination reactions of radical oxidation of triacylglycerols to peroxides and derivatives, the release of hydrogen atoms by phenolic compounds can inhibit the formation of hydroperoxide radicals [41].

**Figure 2:**
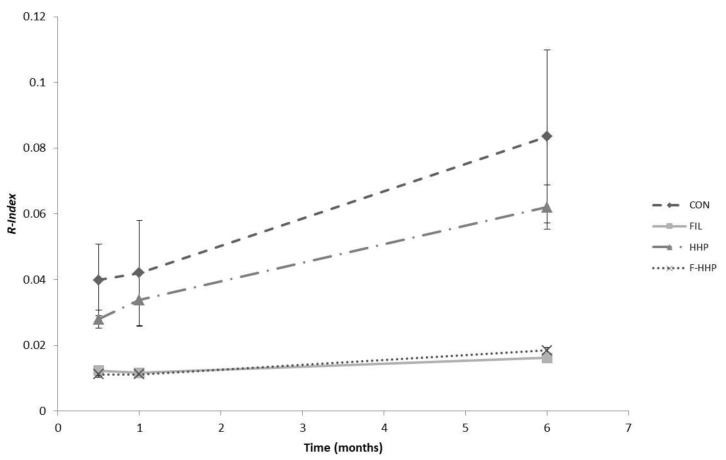
R-index as a function of storage time for the four treatments (CON = veiled and not HHP-treated oil samples; HHP = veiled and HHP-treated oil samples; FIL = filtered and not HHP-treated oil samples; F-HHP = filtered and HHP-treated oil samples). Error bars represent the standard errors.

Our experimental data on the phenolic compound content (Table 4) showed that the secoiridoids in the olive oil samples underwent a clear hydrolytic transformation during storage. This effect can be related to the water content and water activity of the oil samples, without the involvement of microbial activity. Indeed, the 3,4-DHPEA and p-HPEA contents increased during storage and the high level of water activity caused the greatest increase in the veiled oil samples. A hydrolytic increase in 3,4-DHPEA and p-HPEA contents has also been reported in the literature [16,18,42,43]. The R-Index behaviour consistently showed no variations in the hydrolytic status of secoiridoids for the FIL and F-HHP oil samples only (Figure 2).

Our experimental data showed that the secoiridoids also underwent oxidative degradation during storage, but this behaviour was primarily influenced by the absence of the significant radical oxidation of triacylglycerols in all of the olive oil samples. There was no decrease in the total phenolic compound content, or the content of oleuropein, ligstroside and their derivatives (Table 4), conversely to some literature data [16,20] which have shown a decrease in phenolic compound contents due to their antioxidant role. Instead, after 15 days of storage a difference quickly occurred between the contents of oleuropein and its derivatives and 3,4-DHPEA-EDA of the filtered and veiled oil samples (Table 4). The oxidative enzymatic degradation of secoiridoids by endogenous PPO and POD may be involved in the decrease of the above phenolic compounds in the veiled oil samples [44]; this phenomenon may be consistent with both the effect of the water content/water activity and the panellists’ different perceptions of bitterness and pungency between the filtered and veiled oil samples.

## 4. Materials and Methods

### 4.1. Trials

Four trials were carried out during November 2017. Olive fruits of the Frantoio cultivar (approx. 300 kg for each trial) were harvested in the Greve in Chianti area (Florence, Italy) and pressed in an industrial oil mill (Azienda Agricola La Ranocchiaia, Florence, Italy). In brief, the plant was equipped with an olive cleaner, followed by a blade cutter crusher, and 300 kg sealed vertical malaxers. The olive paste was kneaded in the malaxers for 20 min at 18 °C, and extracted by a two-phase horizontal centrifuge (i.e., decanter) with 700 kg/h working capacity. The batches of olive oil were collected at the end of the “decanter” and immediately split as follows: half was immediately filtered, while the other half was left veiled. A filter press equipped with eleven 40 × 40 cm cardboard sheets (CKP V8, Cordenons SpA, Pordenone, Italy) was used. The technical specifications, which were provided by the filter producer, were as follows: weight, 1050 g/m2; thickness, 3.75 mm; nominal cut-off filtration, 12 µm; nominal flow rate, 160 L min^−1^ m^−2^. Then, all of the olive oil samples were bottled in 250 mL transparent PET bottles and half of the bottles underwent HHP treatment. A JBT AvureTM HPP industrial plant (HPP Italia srl, Traversetolo, Parma, Italy) was used. During the HHP treatment, the pressure was increased from atmospheric pressure to a working pressure of 608 MPa in 200 s, the working pressure was maintained for 360 s and then the oil samples were returned, almost instantaneously, to atmospheric pressure. The HHP system remained at 15 °C, which was the minimum temperature to prevent the olive oil from freezing during the decompression stage.

Therefore, the trials resulted in a full factorial design with four specimens: (i) not filtered and not HHP-treated olive oil samples, (ii) filtered and not HHP-treated olive oil samples, (iii) not filtered and HHP-treated olive oil samples, (iv) filtered and HHP-treated olive oil samples.

All of the olive oil samples were transferred to the laboratory; they were immediately analysed to measure some turbidity characterization parameters (i.e., degree of turbidity, water content, water activity, solid particle content, microbial cell count) and then subjected to the storage tests.

### 4.2. Storage Conditions

All of the olive oil samples were stored in a chamber (1.3 × 1.0 × 0.8 m) with the internal walls covered with reflective material. The operating conditions were as follows: constant temperature of 20 °C and light intensity of 1900 lux (Master TL-D 90 Graphica lamp, 35W/390, Philips, Amsterdam, The Netherlands) for 8 h per day. The samples were stored in a random position with adequate space between the transparent bottles, and their positions were changed every two weeks. The olive oil samples were analysed after 15 days, 1 month and 6 months of storage. The analyses at 15 days and 1 months were performed to monitor rapid changes due to microorganisms, while the analysis after 6 months of stored were performed to monitor slow changes in the olive oil chemical profile.

### 4.3. Analyses

The acidity (% oleic acid), peroxide value (meq O_2_ kg^−1^) and UV spectroscopic indices (K_232_, K_270_ and ∆K) were measured according to the official EU method and subsequent amendments [45].

The panel test was carried out according to the official IOC method [46]. The panel was made up of five men and three women, aged from 28 to 57; all of the panellists were non-smokers and had been trained following the official IOC procedure. The panellists worked for the Taste Commission of the Ministero delle Politiche Agricole Alimentari, Forestali e del Turismo (MIPAAAFT - Italian Ministry of Agri-Food and Forestry Policy and Tourism).

The degree of turbidity was measured in nephelometric turbidity units (NTU) using a Hach Model 2100 turbidimeter (Hach, Loveland, CO, USA). About 25 g of the oil samples were put in the standard glass vessel, which was inserted in the closed vessel chamber of the turbidimeter; the degree of turbidity was measured at equilibrium after approx. 1 h.

Water content (% *w*/*w*) was analysed using a Karl Fischer Kit for visual water determination without a titrator (37858 HYDRANAL—Moisture Test Kit, Honeywell Fluka^TM^, Bucharest, Romania). The oil sample (1 mL) was dissolved in previously neutralized HYDRANAL—Solvent E, and the titrating reagent (HYDRANAL—Titrant 5E) was added until the equivalence point was reached.

Water activity (A_w_) was measured using a Rotronic Hygroskop DT hygrometer (Michell Italia Srl, Milan, Italy). The samples (approx. 6.5 mL) were placed in the standard sample cups and the water activity was measured at equilibrium after approx. 12 h.

The solid particle content was measured using the method described by the literature [15]. A 5 g aliquot of filtered oil was vacuum-filtered to saturate Whatman grade 1 filter paper (Merck KGaA, Darmstadt, Germany). The same filter paper was used to filter approx. 30 g of the oil samples and then it was weighed using an analytical balance. The solid particle content was calculated by weighing the difference and quantified in % *w*/*w*.

The microorganisms were enumerated according to the method reported by the literature [47], with some modifications: an aliquot of each sample (i.e., approx. 20 mL) was taken from each bottle in sterile conditions and filtered through a 0.45 µm sterile nitrocellulose membrane. Then, the membrane was transferred into a 50 mL sterile Falcon tube containing 20 mL of sterile physiological solution (NaCl 0.85%), and homogenized using an UltraTurrax (mod. T25 homogenizer, IKA, Milan, Italy). Of each homogenized sample, 200 μL serial dilutions were plated onto a YPD agar medium. After 48–72 h of incubation at 28 °C, the colonies with different morphologies were counted and, for each kind, the cell morphology was observed through a light microscope.

The extraction, identification and determination of the phenolic compounds were performed by RP-HPLC using the official IOC method [48]. Briefly the HPLC apparatus consisted an Agilent 1200 series system (Agilent technoligies, Santa Clara, CA, USA) composed by a quaternary pump equipped with a diode-array detector and autosampler. The analytical conditions were: HPLC column: LiChroCART® 250-4.6 Purospher® STAR RP-18E, 5 µm (250 × 4.6 mm id, Merck KGaA) equipped with a: LiChroCART® 4-4 Purospher® STAR RP-18E, 5 µm pre-column (4 × 4 mm); eluition condition: water 0.2% H_3_PO_4_ (*v*/*v*), methanol, acetonitrile gradient following the official IOC method [48]; injection volume: 20 μL; wavelength: 280 nm. Syringic acid was used as the internal standard; syringic acid and tyrosol were chosen as the external calibration standards to evaluate the relative response factor (i.e., RRF = 4.87). Phenolic compounds were quantified in mg_tyrosol_ kg_oi_^−1^. The total phenolic compound content (mg_tyrosol_ kg_oi_^−1^) was determined asthe sum of the peak areas of phenols recorded at 280 nm.

The R-Index, which was suggested by Fiorini et al. [49] to measure the hydrolytic status of secoiridoids, was also determined as follows:

(1) $$R-Index=\frac{\left(Tyrosol\;content+Hydroxytyrosol\;content\right)}{\left(Tyrosol\;content+Hydroxytyrosol\;content+Secoiridoid\;derivative\;content\right)}$$

The volatile organic compound content of the olive oil was determined according to the method described by the literature [50], using HS-SPME-GC-MS. Analyses were carried out by weighing 4.3 g of the sample and 0.1 g of an internal standard mixture (ISTD MIX) into 20 mL screw-cap vials fitted with a PTFE/silicone septum. After 5 min of equilibrium at 60 °C, the SPME fibre (50/30 µm DVB/CAR/PDMS by Supelco, Darmstadt, Germany) was exposed in the vial headspace for 20 min while being subjected to orbital shaking (500 rpm). Then, the fibre was immediately desorbed for 2 min in a gas chromatograph injection port operating in splitless mode at 260 °C. The identification of the volatile compounds was performed by gas chromatography coupled with quadrupole mass spectrometry using a GC-MS Scientific Trace system (Thermo Fisher, Waltham, MA, USA) equipped with a 30 m × 0.25 mm ID, 0.25 µm DF ZB-FFAP capillary column (Phenomenex, Torrance, CA, USA). The initial column temperature was held at 36 °C for 10 min, then increased to 156 °C at 4 °C/min, then to 260 °C at 10 °C/min, and finally to 250 °C at 10 °C/min, with a hold time of 2 min. Helium was used as the carrier gas at a constant flow of 0.8 mL/min. The temperature of both the ion source and the transfer line was 250 °C. The mass detector was operated in scan mode within a 30–330 Th mass range at 1500 Th/s, with an IE energy of 70 eV. Compounds were identified and quantified (mg/kg) through comparison of their mass spectra and retention times with those of the ISTD MIX. These consisted of the following 11 compounds: 3,4-dimethylphenol, 4-methyl-2-pentanol, hexanoic acid-d_11_, 1-butanol-d_10_, ethyl acetate-d_8_, toluene-d_8_, ethyl hexanoate-d_11_, acetic acid-2,2,2-d_3_, 6-chloro-2-hexanone, 3-octanone and trimethylacetaldehyde.

All the above measurements were carried out in triplicate.

### 4.4. Data Processing

A 3-way ANOVA was performed on each variable to assess the effect of filtration, HHP, storage time and their interactions. The ANOVA showed significant differences (*p* < 0.05) which were studied as follows: first of all, the significant interactions between two variables were studied, then the significance of the three main effects was assessed with a Tukey-HSD post hoc test.

## 5. Conclusions

This study evaluated the EVOO qualitative changes during the storage due to microbial contamination and water content/activity. The microbial contamination level (i.e., mainly yeasts) in presence of a high level of water activity (> 0.6 A_w_) could be related to the formation of volatile aroma compounds, which were responsible for the “fusty” sensory defect. High water activity values could be related to an increase in the degradation rate of LOX compounds; the (*E*)-2-hexanal content decreased, causing a decrease in the “fruity” positive sensory attribute. High water activity values could be also related to an increase in the hydrolytic degradation rate of the phenolic compounds; the 3,4-DHPEA and *p*-HPEA contents increased, causing an increase in the hydrolytic status (R-Index) of the secoiridoids. Thus, microbial contamination and water activity of the oil immediately after extraction could be considered critical control parameters to identify olive oil more prone to degradation during storage.

Since in our study the radical oxidation of the triacylglycerols during storage was negligible in all of the oil samples during 6 months of storage, no relevant potential effects of water activity on the EU legal limits or non-enzymatic oxidative degradation of secoiridoids were evidenced. On the other hand, the absence of the radical oxidation of triacylglycerols could have revealed evidence of the following two degradation phenomena, which would require supplementary studies: (i) microbial activity in the presence of a high level of water activity, which rapidly caused the formation of C_7_, C_8_, C_9_ and C_10_ volatile compounds and the “rancid” sensory defect; (ii) an oxidative enzymatic degradation of secoiridoids in the presence of a high level of water activity, which rapidly caused a decrease in 3,4-DHPEA-EDA and different perceptions by the panellists of the “bitter” and “pungent” positive sensory attributes.

In the end, when an organization wants to produce VEVOO in order to cause a positive visual effect on consumer expectations, the oil turbidity has to be planned and controlled, starting from (i) adjustment of the water content with suitable application of the normal separation treatments after oil extraction by “decanter”; (ii) good manufacturing practices to minimize microbial contamination during the olive oil processing chain.

## Figures and Tables

**Table 1:** Mean values and standard deviations of the turbidity characterization parameters for the four specimens of just processed olive oil samples; CON = veiled and not HHP-treated oil samples; HHP = veiled and HHP-treated oil samples; FIL = filtered and not HHP-treated oil samples; F-HHP = filtered and HHP-treated oil samples. Number of replicates = 4.

Oil Samples	Degree of Turbidity (NTU)	Water Content (%*w*/*w*)	Solid Particle Content (%*w*/*w*)	A_w_	Microbial Cell Count (log UFC/g)
CON	1525 ± 108	0.25 ± 0.09	0.22 ± 0.06	0.76 ± 0.05	4.5 ± 0.2
HHP					0.0 ± 0.0
FIL	17 ± 4	0.05 ± 0.01	0.00 ± 0.00	0.42 ± 0.02	0.0 ± 0.0
F-HHP					0.0 ± 0.0

**Table 2:** Mean values and standard deviation of the panel test sensory attributes for the four specimens of olive oil samples during storage.

Storage Time (Months)	F-HHP			FIL			HHP			CON			*p* F	*p* HP	*p* T	*p* F × T	*p* F × HP	*p* HP × T
	0.5	1	6	0.5	1	6	0.5	1	6	0.5	1	6						
Fruity	5 ± 1 ax	2 ± 1 bx	2 ± 0 bx	5 ± 1 ax	2 ± 1 bx	2 ± 1 bx	3 ± 1 ay	1 ± 1 by	1 ± 1 by	4 ± 1 ay	1 ± 1 by	1 ± 1 by	***	ns	***	ns	ns	ns
Bitter	4 ± 0	2 ± 0	2 ± 1	5 ± 0	2 ± 0	2 ± 1	3 ± 0	2 ± 1	2 ± 1	3 ± 1	2 ± 1	1 ± 1	**	ns	***	*	ns	ns
Pungent	5 ± 0 ax	3 ± 1 bx	2 ± 0 bx	5 ± 1 ax	4 ± 1 bx	3 ± 1 bx	4 ± 0 ay	3 ± 1 by	2 ± 1 by	4 ± 1 ay	2 ± 1 by	1 ± 1 by	*	ns	***	ns	ns	ns
Fusty	nd	nd	nd	nd	Nd	nd	nd	nd	nd	1 ± 0	2 ± 1	2 ± 1	***	*	ns	ns	**	ns
Rancid	nd	nd	nd	nd	Nd	nd	nd	1 ± 0	1 ± 0	nd	2 ± 0	3 ± 1	***	*	***	*	*	*

*, ** and *** indicate significant differences by 3-way ANOVA at *p* < 0.05, *p* < 0.01 and *p* < 0.001, respectively, for the treatments (F = filtration; HP = high pressure), the storage time (T) and their interactions; different letters (i.e., a, b, c for the three storage times; x, y for filtered and unfiltered samples) indicate a statistically significant difference of the main effects with the Tukey HSD post hoc test (*p* < 0.05), while the significant interactions are discussed in the main text. ns = not significant; nd = not detected. CON = veiled and not HHP-treated oil samples; HHP = veiled and HHP-treated oil samples; FIL = filtered and not HHP-treated oil samples; F-HHP = filtered and HHP-treated oil samples. Number of replicates = 4.

**Table 3:** Mean values and standard deviation of the volatile organic compound content for the four specimens of olive oil samples during storage.

Storage Time (Months)	FIL-HHP			FIL			HHP			CON			*p* F	*p* HP	*p* T	*p* F × T	*p* F × HP	*p* HP × T
	0.5	1	6	0.5	1	6	0.5	1	6	0.5	1	6						
Sum of C_5_ compounds	1.7 ± 0.3	1.6 ± 0.3	1.6 ± 0.4	1.8 ± 0.3	1.5 ± 0.4	1.6 ± 0.4	1.5 ± 0.3	1.2 ± 0.1	1.2 ± 0.1	1.4 ± 0.0	1.5 ± 0.1	2.4 ± 1.4	ns	ns	ns	ns	Ns	ns
Sum of C_6_ compounds	33.7 ± 7.1	33.8 ± 3.7	25.9 ± 2.5	35.0 ± 9.2	32.5 ± 3.1	22.9 ± 0.9	30.2 ± 5.8	29.8 ± 2.5	22.8 ± 2.5	34.1 ± 4.2	29.4 ± 7.5	21.7 ± 4.2	ns	ns	*	ns	Ns	ns
E-2-hexenal	30.2 ± 5.2 ax	30.6 ± 5.7 ax	22.6 ± 6.7 bx	31.4 ± 8.7 ax	29.3 ± 11.6ax	19.7 ± 2.3 bx	26.9 ± 3.1 ay	27.1 ± 3.1 ay	20.3 ± 8.8 by	27.5 ± 2.3 ay	19.8 ± 2.0 ay	13.1 ± 0.4 by	**	ns	***	ns	Ns	ns
Sum of microbial metabolite compounds	9.8 ± 2.6	8.3 ± 0.9	8.7 ± 0.6	9.7 ± 2.4	7.4 ± 1.1	9.2 ± 0.9	8.8 ± 2.0	6.6 ± 0.5	8.2 ± 0.5	7.6 ± 0.9	19.2 ± 9.5	35.2 ± 10.2	ns	ns	ns	ns	*	ns
Sum of C_7_, C_8_, C_9_ and C_10_ compounds	1.7 ± 0.1	1.7 ± 0.2	2.2 ± 0.3	1.4 ± 0.1	1.6 ± 0.2	2.2 ± 0.2	1.2 ± 0.1	1.7 ± 0.1	1.9 ± 0.1	4.2 ± 2.1	1.9 ± 0.1	1.8 ± 0.1	**	**	ns	ns	**	ns

*, ** and *** indicate significant differences by 3-way ANOVA at *p* < 0.05, *p* < 0.01 and *p* < 0.001, respectively, for the treatments (F = filtration; HP = high pressure), the storage time (T) and their interaction; different letters (i.e., a, b, c for the three storage times; x, y for filtered and unfiltered samples) indicate a statistically significant difference of the main effects with the Tukey HSD post hoc test (*p* < 0.05), while the significant interactions are discussed in the main text. ns = not significant. CON = veiled and not HHP-treated oil samples; HHP = veiled and HHP-treated oil samples; FIL = filtered and not HHP-treated oil samples; F-HHP = filtered and HHP-treated oil samples. All concentrations are expressed in mg/kg. Number of replicates = 4.

**Table 4:** Mean values and standard deviation of the phenolic compound content for the four specimens of olive oil samples during storage.

Storage Time (Months)	FIL-HPP			FIL			HHP			CON			*p* F	*p* HP	*p* T	*p* F × T	*p* F × HP	*p* HP × T
	0.5	1	6	0.5	1	6	0.5	1	6	0.5	1	6						
Sum of oleuropein and its derivatives	333 ± 22 a	306 ± 40 a	309 ± 29 a	346 ± 9 a	317 ± 26 a	290 ± 25 a	218 ± 42 b	228 ± 37 b	248 ± 56 b	229 ± 45 b	229 ± 44 b	241 ± 58 b	***	ns	ns	ns	ns	ns
3,4-DHPEA-EDA	122 ± 14 a	135 ± 16 a	126 ± 25 a	123 ± 7 a	112 ± 17 a	131 ± 15 a	65 ± 22 b	71 ± 23 b	64 ± 21 b	67 ± 24 b	68 ± 11 b	60 ± 22 b	***	ns	ns	ns	ns	ns
Hydroxytyrosol	1 ± 0	2 ± 0	3 ± 0	1 ± 0	1 ± 0	3 ± 0	6 ± 1	6 ± 2	15 ± 3	8 ± 3	7 ± 4	17 ± 3	***	ns	***	***	ns	ns
Sum of ligstroside and its derivatives	106 ± 15	109 ± 15	128 ± 23	118 ± 22	109 ± 17	116 ± 13	124 ± 23	113 ± 22	135 ± 25	125 ± 26	115 ± 29	126 ± 28	ns	ns	ns	ns	ns	ns
p-HPEA-EDA	71 ± 6	68 ± 8	73 ± 14	73 ± 5	67 ± 12	75 ± 9	70 ± 12	65 ± 13	70 ± 14	74 ± 14	68 ± 21	64 ± 17	ns	ns	ns	ns	ns	ns
Tyrosol	2 ± 0	2 ± 0	3 ± 0	2 ± 0	2 ± 0	3 ± 0	2 ± 1	3 ± 0	6 ± 1	3 ± 1	4 ± 2	9 ± 6	***	ns	***	***	ns	ns
Total phenolic compounds	448 ± 20	474 ± 42	484 ± 46	479 ± 19	468 ± 48	469 ± 37	418 ± 66	429 ± 52	481 ± 81	434 ± 68	420 ± 61	472 ± 72	ns	ns	ns	ns	ns	ns
R-Index (10^−2^)	1 ± 0	1 ± 0	2 ± 0	1 ± 0	1 ± 0	1 ± 0	3 ± 1	3 ± 2	6 ± 1	4 ± 2	4 ± 3	8 ± 5	***	ns	***	***	ns	ns

*, ** and *** indicate significant differences by 3-way ANOVA at *p* < 0.05, *p* < 0.01 and *p* < 0.001, respectively, for the treatments (F = filtration; HP = high pressure), the storage time (T) and their interaction; different letters (i.e., a, b, c for the three storage times; x, y for filtered and unfiltered samples) indicate a statistically significant difference of the main effects with the Tukey HSD post hoc test (*p* < 0.05), while the significant interactions are discussed in the main text. ns = not significant; nd = not detected. CON = veiled and not HHP-treated oil samples; HHP = veiled and HHP-treated oil samples; FIL = filtered and not HHP-treated oil samples; F-HHP = filtered and HHP-treated oil samples; 3,4-DHPEA-EDA = dialdehydic form of decarboxymethyl elenolic acid linked to hydroxytyrosol; p-HPEA-EDA = dialdehydic form of decarboxymethyl ligstroside aglycones. All concentrations are expressed in mg/kg. Number of replicates = 4.

## Supplementary Materials

The following are available online at https://www.mdpi.com/1420-3049/25/2/420/s1, Table S1: report the mean and standard deviation of free fatty acids content, peroxide value and UV indexes for the four specimens of olive oil samples during storage, Table S2: Groups of the volatile organic compounds identified and measured in the oil samples.

## Abbreviations

EVOO	Extra virgin olive oil
VEVOO	veiled extra virgin olive oil
FEVOO	filtered extra virgin olive oil
HHP	olive oil treated with high hydrostatic pressure
FIL	filtered olive oil
F-HPP	olive oil filtered and treated with HHP
CON	not treated olive oil
LOX	Lipoxygenase
3,4-DHPEA-EDA	dialdehydic form of decarboximethyl elenolic acid linked to hydroxytyrosol
3,4-DHPEA-EA	oleuropein aglycones
3,4-DHPEA	Hydroxytyrosol
*p*-HPEA-EDA	dialdehydic form of decarboxymethyl ligstroside aglycones
*p*-HPEA-EA	ligstroside aglycones
*p*-HPEA	Tyrosol
LOX	Lipoxygenase
YPD	Yeast Extract–Peptone–Dextrose Broth
