# Supplementary material for: Understanding Olive Oil Stability Using Filtration and High Hydrostatic Pressure
Source: Molecules. 2020 Jan 20;25(2):420. doi: 10.3390/molecules25020420 (PMC7024224; doi:10.3390/molecules25020420)
Supplement: Supplementary file 1 [file molecules-25-00420-s001.pdf]

TABLE S1. Mean and standard deviation of free fatty acids content (FFA), peroxide value and UV indexes for the four specimens of olive oil samples during storage. Different letters (i.e. a, b for filtration and x, y for storage time) indicate a statistically significant difference with the Tukey HSD post hoc test ($p<0.05$). CON = veiled and not HHP-treated oil samples; HHP = veiled and HHP-treated oil samples; FIL = filtered and not HHP-treated oil samples; F-HHP = filtered and HHP-treated oil samples.

Storage time (months)	*F-HHP*			*FIL*			*HHP*			*CON*		
	0.5	1	6	0.5	1	6	0.5	1	6	0.5	1	6
FFA (% oleic acid)	0.19 ± 0.03	0.21 ± 0.01	0.19 ± 0.04	0.20 ± 0.03	0.19 ± 0.04	0.19 ± 0.04	0.21 ± 0.04	0.16 ± 0.02	0.23 ± 0.06	0.21 ± 0.02	0.21 ± 0.03	0.27 ± 0.02
K_{232}	1.7 ± 0.1 b	1.8 ± 0.1b	1.71 ± 0.05 b	1.75 ± 0.04 b	1.7 ± 0.1 b	1.74 ± 0.09 b	1.58 ± 0.03 a	1.63 ± 0.06 a	1.6 ± 0.1 a	1.57 ± 0.06 a	1.61 ± 0.01 a	1.59 ± 0.02 a
K_{270}	0.13 ± 0.01	0.15 ± 0.01	0.14 ± 0.01	0.13 ± 0.01	0.15 ± 0.01	0.15 ± 0.01	0.10 ± 0.01	0.13 ± 0.01	0.130 ± 0.008	0.100 ± 0.008	0.14 ± 0.01	0.130 ± 0.005
ΔK	0.004 ± 0.001 ax	0.003 ± 0.003 ay	0.003 ± 0.000 ay	0.004 ± 0.000 ax	0.003 ± 0.001 ay	0.003 ± 0.001 ay	0.003 ± 0.001 bx	0.002 ± 0.001 by	0.002 ± 0.000 by	0.003 ± 0.001 bx	0.002 ± 0.000 by	0.001 ± 0.000 by
Peroxide value (meq_{O2}/kg)	4.6 ± 0.9 bx	5.8 ± 0.4 by	6.4 ± 0.4 by	4.3 ± 0.4 bx	5.3 ± 0.8 by	5.7 ± 0.8 by	3.9 ± 1.0 ax	4.6 ± 0.5 ay	4.9 ± 0.8 ay	4.4 ± 0.3 ax	4.6 ± 0.9 ay	4.3 ± 0.5 ay

TABLE S2. Groups of the volatile organic compounds identified and measured in the oil samples.

**C5 compounds**	**C6 compounds**	**Microbial metabolite compounds**	**C7-C8-C9-C10 compounds**
3-pentanone	hexanal	methanol	heptane
pentanal	E-2-hexenal	propanol	octane
1-penten-3-one	Z-3-hexenal	methyl acetate	heptanal
2-pentanol	hexyl acetate	isobutanol	octanal
E-2-pentenal	E-2-hexenyl acetate	ethyl acetate	2-octanone
1-penten-3-ol	Z-3-hexenyl acetate	2-butanone	2-heptanol
1-pentanol	1-hexanol	methyl propionate	E-2-heptenal
E-2-penten-1-ol	E-3-hexen-1-ol	butanal-2-methyl	5-hepten-2-one-6-methyl
Z-2-penten-1-ol	Z-3-hexen-1-ol	butanal-3-methyl	2-nonanone
	E-2-hexen-1-ol	ethanol	nonanal
	Z-2-hexen-1-ol	ethyl propanoate	2,4-hexadienal
		R-2-butanol	2,4-heptadienal
		butanoic acid ethyl ester	decanal
		acetic acid butyl ester	E-2-decenal
		2-methyl butanol	2,4-nonadienal
		3-methyl butanol	2,4-decadienal
		2-octanol	
		E-2-octenal	
		1-octen-3-ol	
		acetic acid	
		1-octanol	
		butanoic acid	
		propanoic acid	
		phenol-2-methoxy	
		phenylethyl alcohol	
		phenol	
		phenol-4-ethyl-2-methoxy	
		4-ethyl phenol	
